# Ultrasound-Guided Percutaneous Gastrostomy for Decompression of the Obstructed Excluded Stomach Following Roux-en-Y Gastric Bypass: A Case Report

**DOI:** 10.7759/cureus.112375

**Published:** 2026-07-09

**Authors:** Alisher Tashbayev, Sean Liddle, Sophie Short

**Affiliations:** 1 Radiology, Whangarei Hospital, Te Whatu Ora, Whangarei, NZL; 2 Surgery, Whangarei Hospital, Te Whatu Ora, Whangarei, NZL

**Keywords:** altered postoperative anatomy, bariatric surgery, excluded stomach, gastric remnant, interventional radiology, percutaneous decompression, roux-en-y gastric bypass, ultrasound-guided gastrostomy

## Abstract

Access to the excluded stomach following Roux-en-Y gastric bypass (RYGB) can be technically challenging because of altered postoperative anatomy. We present the case of a 45-year-old woman with a history of RYGB and subsequent surgery for small bowel ischemia secondary to an internal hernia who developed marked distension of the excluded stomach, consistent with obstruction at the duodenojejunal anastomosis. Pre-procedural computed tomography (CT) demonstrated severe distension of the gastric remnant and was used to assess its relationship to adjacent bowel loops and identify a safe percutaneous access route. The patient underwent ultrasound-guided percutaneous gastrostomy in the operating theater under general anesthesia. Following gastropexy, guidewire placement, and tract dilation, an 18-Fr gastrostomy tube was successfully inserted, resulting in immediate decompression with aspiration of approximately 2 L of gastric contents. At the two-month follow-up, the patient had experienced significant clinical improvement, and repeat CT demonstrated resolution of the obstruction. This case illustrates that ultrasound-guided decompressive gastrostomy may represent a useful therapeutic option in carefully selected patients with complex postsurgical anatomy when the excluded stomach is distended and sonographically accessible.

## Introduction

**Figure 1:**
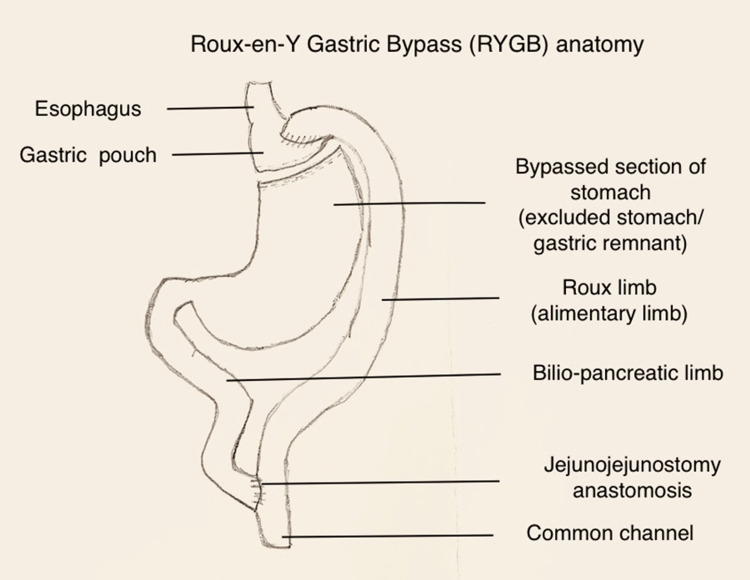
Schematic illustration of Roux-en-Y gastric bypass (RYGB) anatomy This diagram shows the creation of a small gastric pouch, the Roux (alimentary) limb, the biliopancreatic limb, and the common channel following jejunojejunostomy. Food passes from the gastric pouch through the Roux limb, while biliopancreatic secretions travel separately through the biliopancreatic limb before mixing within the common channel. Image Credit: This image was hand-drawn Alisher Tashbayev and then digitized by photographing the original drawing. The labels and annotations were added using the Markup tools in Preview (macOS; Apple, Cupertino, CA, USA). No generative AI tools were used in the creation of this figure.

Roux-en-Y gastric bypass (RYGB) creates a small gastric pouch that is anastomosed to the jejunum, forming the Roux (alimentary) limb. The bypassed stomach, duodenum, and proximal jejunum form the biliopancreatic limb, which carries biliopancreatic secretions distally to the jejunojejunostomy, where they mix with enteric contents in the common channel (Figure 1). This altered anatomy renders the excluded stomach inaccessible by conventional upper gastrointestinal endoscopy and may complicate subsequent diagnostic and therapeutic interventions.

Access to the excluded stomach may occasionally be required for gastric decompression, enteral access, endoscopic evaluation, or management of postoperative complications. However, percutaneous access can be technically challenging because of altered bowel configuration, variable positioning of the gastric remnant, interposed bowel loops or other viscera, and postoperative adhesions [1-3].

Percutaneous radiologic gastrostomy (PRG) is an established minimally invasive technique for enteral feeding and gastric decompression, with high technical success rates and lower morbidity than surgical approaches [4]. Traditionally, fluoroscopic guidance has been used for PRG placement, while computed tomography (CT)-guided and endoscopic ultrasound-assisted techniques have also been described in patients with complex anatomy [2,3]. More recently, ultrasound-assisted gastric access has gained interest because it provides real-time visualization of the stomach and adjacent structures and may reduce the risk of inadvertent bowel injury [5,6]. Despite increasing experience with image-guided gastrostomy, reports specifically describing ultrasound-guided decompressive gastrostomy of the excluded stomach in patients with post-RYGB anatomy remain limited [6]. We describe a case in which careful pre-procedural CT planning and real-time ultrasound guidance facilitated safe decompressive access to a markedly distended excluded stomach.

## Case presentation

We present the case of a 45-year-old woman with a history of RYGB performed for morbid obesity in Mexico in 2024 (Figure 1).

Eleven months after her index surgery, the patient presented critically ill with obstruction of a long segment of the biliopancreatic (BP) limb, accompanied by signs of bowel ischemia. She underwent emergency laparotomy, which revealed an internal hernia through Petersen's defect with infarction of the BP limb, necessitating bowel resection. A planned second-look laparotomy was performed the following day to complete intestinal reconstruction. During the second-stage procedure, intestinal continuity was restored by creating two new small bowel anastomoses. The biliopancreatic stump was anastomosed to the common channel, followed by reconstruction of the Roux limb 20 cm distally, thereby re-establishing bowel continuity. After a period of recovery in the intensive care unit (ICU) followed by transfer to the surgical ward, the patient was discharged in good clinical condition.

**Figure 2:**
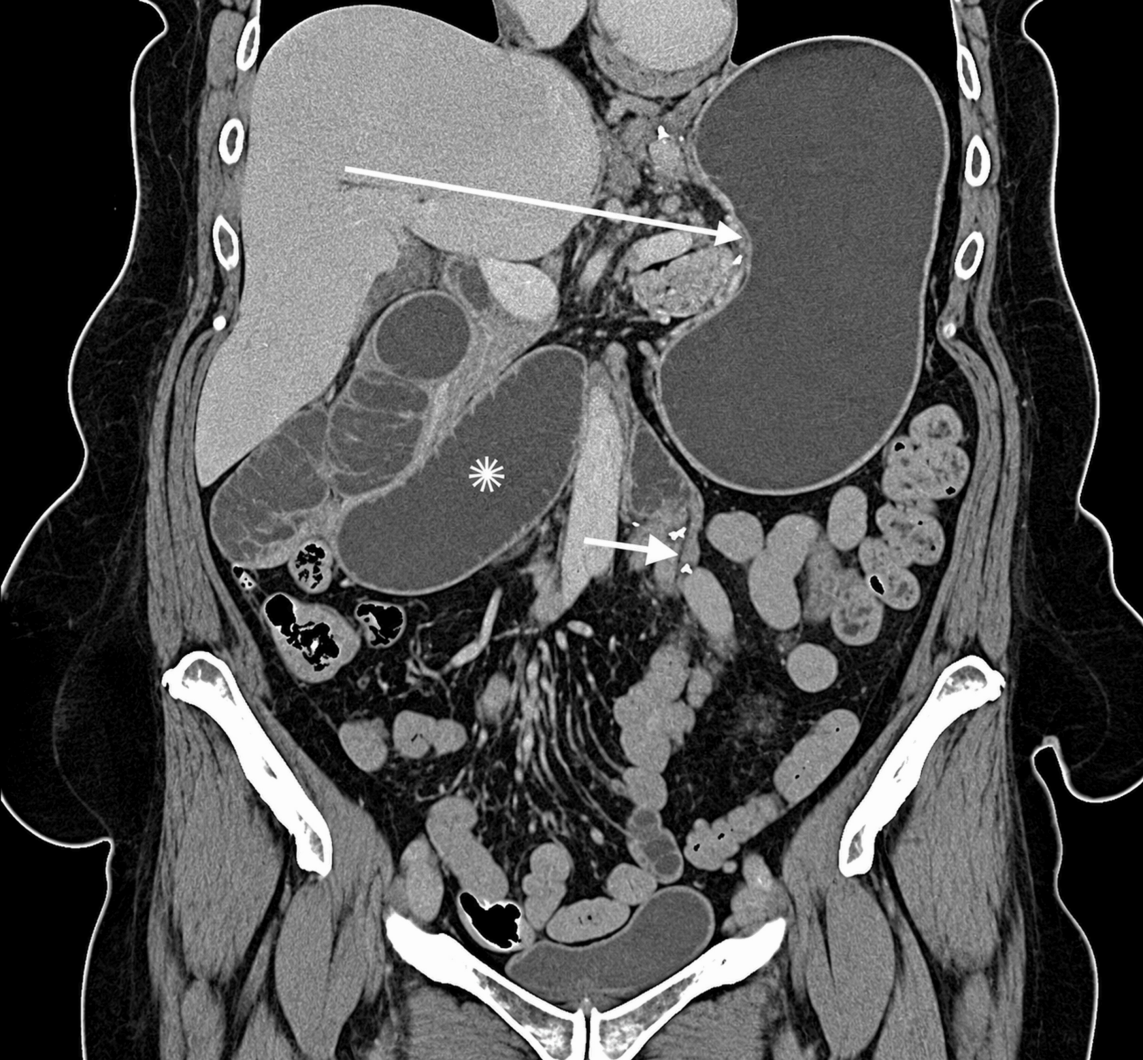
Pre-procedural coronal CT demonstrating gastric remnant distension Coronal CT image demonstrating marked distension of the excluded stomach (white arrow) and dilatation of the biliopancreatic limb (asterisk) secondary to stenosis of the proximal jejunojejunal anastomosis (short white arrow) in a patient with prior Roux-en-Y gastric bypass.

Two months later, she presented again with progressive nausea and worsening upper abdominal pain. Clinical and radiological evaluation demonstrated stenosis at the proximal jejunojejunal anastomosis of the biliopancreatic limb. CT revealed marked distension of the gastric remnant (excluded stomach), with significant fluid and gas accumulation (Figure 2).

**Figure 3:**
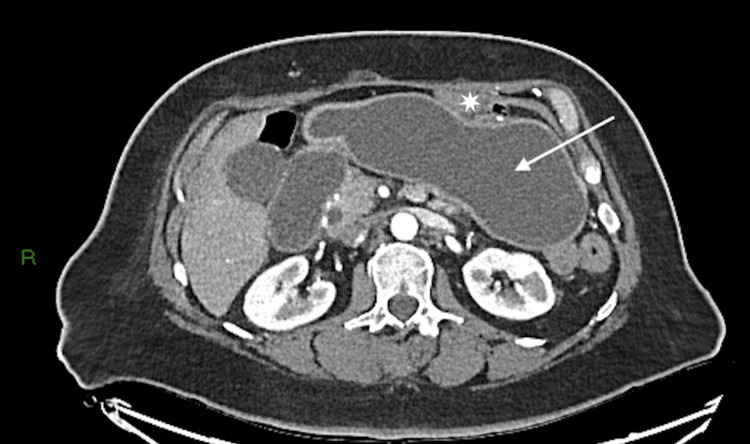
Pre-procedural axial CT demonstrating gastric remnant anatomy Axial contrast-enhanced CT image showing the relationship of the distended gastric remnant (white arrow) and the Roux limb (asterisk) to the anterior abdominal wall.

Given the patient's altered postoperative anatomy and the need for gastric decompression, a multidisciplinary decision was made to proceed with image-guided percutaneous gastrostomy. The procedure was performed in the operating theater under general anesthesia. Pre-procedural CT was carefully reviewed to delineate the postoperative anatomy, assess the relationship of the excluded stomach to adjacent bowel loops, and identify a safe percutaneous access route (Figure 3).

Procedure

Ultrasound examination demonstrated a markedly distended gastric remnant that was accessible from the anterior abdominal wall. Real-time sonographic guidance was used to identify the excluded stomach and avoid adjacent structures, including bowel loops and the Roux limb.

Following sterile preparation and local anesthetic infiltration, the gastric remnant was punctured under ultrasound guidance. Gastrostomy was performed using the PEG Pull Kit MIC® 24 Fr (Avanos Medical, Inc., Alpharetta, GA, USA). Gastropexy was performed using anchoring devices before guidewire placement and tract dilatation, after which an 18 Fr gastrostomy tube was successfully inserted into the excluded stomach. Correct intraluminal positioning was confirmed by contrast injection. Approximately 2 L of gastric contents were aspirated immediately after tube placement, resulting in effective decompression.

**Figure 4:**
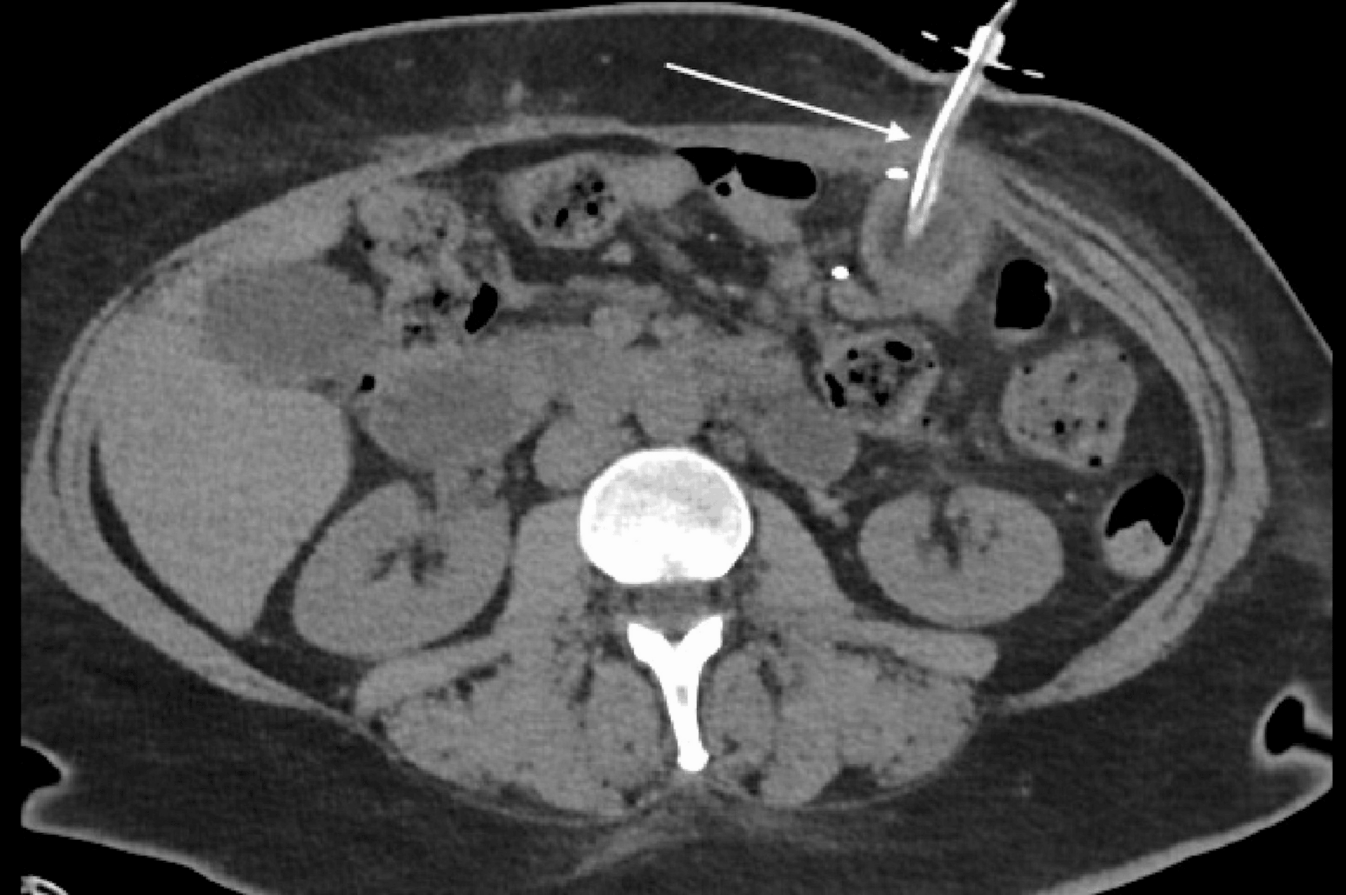
Post-procedural axial CT following percutaneous gastrostomy Post-procedural axial CT image demonstrating decompression of the excluded stomach after gastrostomy tube placement (white arrow).

**Figure 5:**
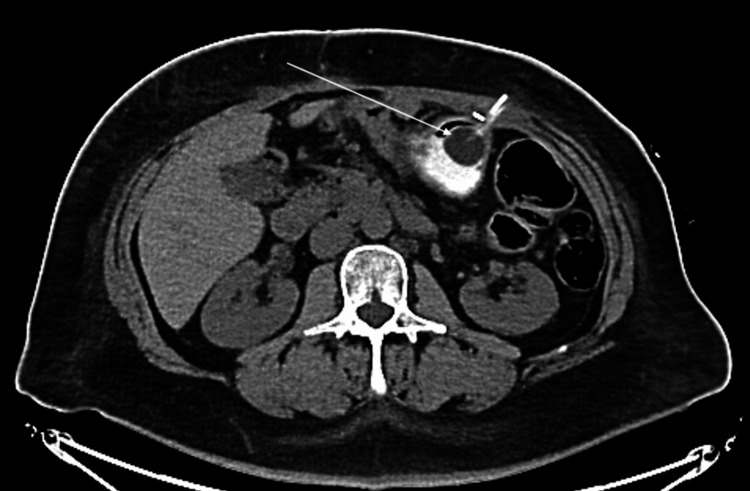
Follow-up CT confirming intraluminal gastrostomy tube position Follow-up CT image demonstrating gastrostomy tube position (white arrow) within the excluded stomach with contrast confirmation of intraluminal placement.

At the planned outpatient follow-up visit two months later, the patient had improved markedly and had returned to her premorbid level of function. Repeat CT demonstrated resolution of the gastric remnant distension (Figures 4, 5). Improvement at the site of stenosis was inferred from the patient's clinical improvement, reassuring imaging findings, and a marked reduction in gastrostomy tube output documented by the patient. Following sustained clinical improvement, the gastrostomy tube was removed.

## Discussion

Percutaneous access to the excluded stomach following RYGB is uncommon but clinically important. Access may be required for gastric decompression, enteral nutrition, management of leaks, or facilitation of endoscopic procedures [1-3]. However, the altered postoperative anatomy may make conventional fluoroscopic or endoscopic approaches technically challenging.

Shaikh et al. described a large single-center experience with image-guided percutaneous gastrostomy of the excluded stomach following RYGB, demonstrating high technical success rates [1]. Other authors have reported CT-guided access to the excluded stomach and the combined use of fluoroscopic techniques in patients with complex postoperative anatomy [2,3]. Ultrasound-guided gastric access has also been described as a safe and feasible technique in carefully selected patients undergoing gastrostomy placement [5,6].

In the present case, pre-procedural CT played a critical role in defining the postoperative anatomy and identifying a safe access route. Unlike many previously reported techniques that relied primarily on fluoroscopic or CT guidance, the markedly distended excluded stomach was readily visualized on ultrasound, allowing real-time image-guided puncture while avoiding adjacent bowel loops and the Roux limb.

This case should not be interpreted as suggesting that ultrasound guidance is universally preferable for patients with altered post-RYGB anatomy. Rather, it demonstrates that ultrasound-guided decompressive gastrostomy may be a feasible option in carefully selected patients in whom the excluded stomach is sufficiently distended and sonographically accessible. The ability to visualize adjacent bowel loops and the Roux limb in real time may provide an additional procedural safety advantage in selected cases.

Gastropexy was performed before tract dilatation to stabilize the stomach against the anterior abdominal wall and minimize the risk of leakage or tube displacement during catheter insertion [4]. Immediate decompression following tube placement confirmed both technical success and therapeutic effectiveness.

## Conclusions

Ultrasound-guided percutaneous decompressive gastrostomy may represent a useful treatment option in selected patients with complex post-RYGB anatomy when the excluded stomach is distended and safely accessible. Careful pre-procedural CT assessment, thorough understanding of the altered surgical anatomy, and real-time ultrasound guidance are important procedural considerations that may facilitate safe and successful access.
